# Biorefinery of Brewery Spent Grain by Solid-State Fermentation and Ionic Liquids

**DOI:** 10.3390/foods11223711

**Published:** 2022-11-18

**Authors:** David Outeiriño, Iván Costa-Trigo, Ricardo Pinheiro de Souza Oliveira, Nelson Pérez Guerra, José Manuel Salgado, José Manuel Domínguez

**Affiliations:** 1 Industrial Biotechnology and Environmental Engineering Group “BiotecnIA”, Chemical Engineering Department, Campus Ourense, University of Vigo, 32004 Ourense, Spain; 2 Biochemical and Pharmaceutical Technology Department, Faculty of Pharmaceutical Sciences, Sao Paulo University, Av. Prof Lineu Prestes, 580, Bl 16, Sao Paulo 05508-900, Brazil; 3 Department of Analytical and Food Chemistry, Faculty of Sciences, Campus Ourense, University of Vigo, As Lagoas s/n, 32004 Ourense, Spain

**Keywords:** biorefinery, brewery spent grain, ionic liquids, *Aspergillus brasiliensis*, *Trichoderma reesei*

## Abstract

Novel environmentally friendly pretreatments have been developed in recent years to improve biomass fractionation. Solid-state fermentation (SSF) and treatment with ionic liquids show low environmental impact and can be used in biorefinery of biomass. In this work, these processes were assessed with brewery spent grain (BSG). First, BSG was used as a substrate to produce cellulases and xylanases by SSF with the fungi *Aspergillus brasiliensis* CECT 2700 and *Trichoderma reesei* CECT 2414. Then, BSG was pretreated with the ionic liquid [N_1112_OH][Gly] and hydrolyzed with the crude enzymatic extracts. Results showed that SSF of BSG with *A. brasiliensis* achieved the highest enzyme production; meanwhile, the pretreatment with ionic liquids allowed glucan and xylan fractions to increase and reduce the lignin content. In addition, a mixture of the extracts from both fungi in a ratio of 2.5:0.5 *Aspergillus*/*Trichoderma* (*v*/*v*) efficiently hydrolyzed the BSG previously treated with the ionic liquid [N_1112_OH][Gly], reaching saccharification percentages of 80.68%, 54.29%, and 19.58% for glucan, xylan, and arabinan, respectively. In conclusion, the results demonstrated that the BSG biorefinery process developed in this work is an effective way to obtain fermentable sugar-containing solutions, which can be used to produce value-added products.

## 1. Introduction

Lignocellulosic biomass is the cheapest and most abundant renewable carbon source that exists. It is generally composed of cellulose (40–50%), hemicellulose (20–30%), and lignin (10–15%), as well as other minor polymers, proteins, extracts, and inorganic matter. This composition varies substantially depending on the biomass source and its origin, climate, harvesting season, processing, and storage conditions to which it has been subjected [1]. These materials have gained importance in the framework of the biorefinery, which aims to efficiently use biomass to obtain fuels, energy, and other bioproducts with high added value in a sustainable way [2].

There are several suitable raw materials for these processes, but the residues from agro-industrial activities are especially interesting because of their appropriate composition. Furthermore, the valorization of these residues has economic and environmental advantages, increasing value in agricultural work and the rural environment [3]. The generation of biomass wastes in the agriculture sector is estimated to be 5 × 10^9^ tons per year [4]. Most of these wastes are not treated and end up being disposed of by burning or dumped in landfills, which is harmful to the environment [5]. We highlight brewery spent grain (BSG), the main byproduct of the brewing industry which, due to its high generation (39 million tons per year in the world), is usually classified as waste due to the existence of an excess with respect to the demand for its main use in animal feed. The possibilities of valorization of BSG in the field of biotechnology have already been reported, but none have been implemented at an industrial level [6,7,8].

Enzymatic hydrolysis is considered a fast, effective, safe, and ecological process that allows the biochemical conversion of biomass polysaccharides into their constituent monomers, without generating byproducts. As a result, this process can be integrated into a biorefinery concept for subsequent microbial fermentation [9]. Cellulose is a linear and unbranched homopolymer of glucose units linked by (β1→4) glycosidic bonds, while hemicellulose is a heterogeneous and branched polysaccharide made up mainly of pentoses (xylose and arabinose) and hexoses (glucose, galactose, and mannose) [10]. To efficiently decompose these polymers, an enzymatic cocktail containing cellulases, hemicellulases, and other accessory enzymes is needed [11]. The global rate of the process is influenced by the structural characteristics of the biomass and the origin of the enzymes [12]. However, this stage can be a limitation for the biorefinery process given the high cost and thermostability of enzymes available on the market, as well as the low conversion yields due to the characteristics of lignocellulosic materials [10,13].

Filamentous fungi are among the most efficient extracellular cellulases producers [14], mainly those belonging to the *Aspergillus* and *Trichoderma* genera. In this sense, solid-state fermentation (SSF) has proven to be an appropriate technique to produce these enzymes, since it simulates the natural habitat of these microorganisms. SSF of wastes, such as BSG used as a source of carbon and energy, can lead to the production of high concentrations of enzymes with high volumetric productivities at low production costs [10,11,13,15].

Most of the cellulases and hemicellulases used in industrial applications come from filamentous fungi, capable of secreting enzyme complexes with a high concentration of these enzymes. Fermentations with fungi of the genera *Aspergillus* and *Trichoderma* stand out in the production of enzymes for the degradation of lignocellulosic biomass [16]. Within the genus *Aspergillus*, the use of those that make up the Nigri section are highlighted for their industrial importance in both food mycology and biotechnology. In this sense, *Aspergillus brasiliensis* is an excellent producer of cellulases, xylanases, and accessory enzymes, such as feruloyl esterases [17,18,19]. On the other hand, *Trichoderma reesei* is widely used in biorefinery processes due to its high cellulases productivity [20].

The complex composition and structure of lignocellulosic materials make them highly resistant to microbial and enzymatic degradation, so it is necessary to carry out a previous treatment to facilitate the action of enzymes on the biomass. To solve this problem, various pretreatments have been studied to remove components that prevent hydrolysis, such as lignin, and modify the structure of the material, reducing the crystallinity of cellulose and increasing the contact surface with the creation of pores [21].

In this sense, various physical, chemical, and biological alternatives have been studied. The most common treatments are based on the use of diluted acids or alkalis and organic solvents, which imply an extra danger and toxicity [22]. Ionic liquids (IL) appear as an interesting alternative due to their high stability and good capacity to dissolve lignocellulosic biomass (completely) or its components (selectively). However, some ILs based on the imidazolium cation, which have proven to be very effective in delignification treatments, were found to be toxic and persistent in the aquatic environment and in the soil [23]. Due to this, there is a growing interest in using ILs based on amino acids, since they are biodegradable and nontoxic, and highly effective in the delignification of biomass [24].

Cholinium-based ILs have offered good results in terms of delignification, being able to cause structural changes, including reduction of crystallinity and formation of pores, which favor enzymatic hydrolysis. In addition, they have turned out to be recyclable and can be reused between five and eight times, depending on the IL, the treatment, and the material to be treated, thus reducing the cost of the process [25,26].

The sugars released after hydrolysis can be used as a carbon source in different biological processes to produce biomolecules and high value-added products [27].

The functional foods market has experienced rapid growth in recent years, with an expected annual increase of 8% [28]. Functional foods are those that have a positive impact on the health, physical performance, or mood of the individual, in addition to their nutritional value. Recent research has paid attention to bioactive compounds and their use to obtain functional foods since these compounds can exhibit antimicrobial, antioxidant, antimutagenic, antiallergic, and anti-inflammatory activities [29].

Probiotics hold promise in this field; however, the low concentration of biologically active compounds derived from traditional probiotics (live probiotic microorganisms) are ineffective under in vivo conditions. Thus, postbiotics, non-viable bacterial products, or metabolic products of microorganisms that are active in the host are presented to prepare these foods. The various postbiotic molecules include metabolic byproducts of live probiotic bacteria, such as cell-free supernatant, vitamins, organic acids, short-chain fatty acids, secreted proteins/peptides, bacteriocins, neurotransmitters, secreted biosurfactants, amino acids, flavonoid-derived postbiotics, etc. [28]. These metabolites, despite having significant potential in the food industry, do not have a wide application in it, due to problems of uneconomic or non-competitive costs for their production compared to their counterparts from plants or chemical products. The production of these compounds from economic and sustainable processes, using agro-industrial residues as in this case, are presented as an alternative [30].

This study focuses on a biorefinery process with BSG as the basis to obtain sugar rich solutions that can be used to produce biomolecules of industrial interest. First, the production of cellulolytic enzymes with *Aspergillus brasiliensis* CECT 2700 and *Trichoderma reesei* CECT 2414 by SSF was studied. Second, the enzymatic hydrolysis of BSG previously treated with IL [N_1112_OH][Gly] was assayed. For the latter purpose, enzymatic extracts previously obtained were used to perform a satisfactory hydrolysis.

## 2. Materials and Methods

### 2.1. Materials

Brewery spent grain (BSG), from the artisanal production of beer, was kindly provided by Letra (Vila Verde, Braga, Portugal). BSG with approximately 80% water content, was dried at 50 °C up to 10% humidity, grinded in an electric shredder MTD 220E (Saarbrücken, Germany), milled to dust with an IKA^®^ Werke model M 20 mill (Staufen, Germany), sieved to size below 5 mm, homogenized in a single lot, and stored at 4 °C before experimentation.

### 2.2. Microorganisms

*Aspergillus brasiliensis* CECT 2700 and *Trichoderma reesei* CECT 2414 were grown on potato dextrose agar (PDA) slants at 30 °C in a moisture-saturated atmosphere for 7 days and stored at 4 °C before use for enzyme production. Both strains were obtained from the Spanish Type Culture Collection (CECT, Valencia, Spain).

### 2.3. Pretreatment with Ionic Liquid

BSG was treated with the ionic liquid (IL) [N_1112_OH][Gly] as described by Outeiriño et al. [31]. Briefly, BSG was treated at 90 °C, during 16 h, and a solid loading of 5 wt% with the ionic liquid (IL) [N_1112_OH][Gly]. Then, a water-acetone solution (1:1) was added which led to the precipitation of a carbohydrate rich material (CRM) with a low lignin content. The process was repeated for 5 successive cycles and the resulting CRMs were mixed.

### 2.4. Solid-State Fermentation (SSF)

The enzymes were produced in 250 mL Erlenmeyer flasks containing 5 g (dry weight) BSG moistened (1:2.5 *w*/*v*) with a solution of mineral salts (1.3 g/L (NH_4_)_2_SO_4_, 5.0 g/L NaNO_3_, 4.5 g/L KH_2_PO_4_, and 3 g/L yeast extract). The mixture was sterilized in autoclave (Trade Raypa SL, Terrassa, Barcelona, Spain) at 100 °C for 60 min. After cooling, each flask was inoculated with a suspension of spores of 1 × 10^6^ spores/g dry BSG by adding 0.1% peptone water with 0.05% Tween 80 to slant cultures.

SSF was performed at 30 °C in a water-saturated atmosphere for 6 days. All experiments were conducted in triplicate.

### 2.5. Enzymes Extraction

Crude extracts were obtained by adding citrate buffer pH 4.8 to SSF media (10 mL/g dry BSG) and incubated for 1 h, 200 rpm at 30 °C. Solids were separated from the extract by centrifugation at 2755× *g* for 15 min (Ortoalresa, Consul 21, EBA 20, Madrid, Spain) and filtered. Subsequently, the enzymatic extracts were frozen at −20 °C to be stored until further analysis [32].

### 2.6. Enzymatic Hydrolysis

The enzymatic hydrolysis was carried out on CRMs, with the enzymatic cocktails from *A. brasiliensis* and *T. reesei* obtained after SSF. Enzymatic hydrolysis was performed in 100 mL Erlenmeyer flasks at 50 °C and 150 rpm during 144 h to ensure complete reaction. To obtain the best hydrolysis conditions, samples were withdrawn each 24 h and immediately centrifuged at 9503× *g* for 10 min, to remove solids. The liquid phase (hydrolyzate) was heated for 5 min on a boiling water bath to stop the reaction. Glucose, cellobiose, xylose, and arabinose in hydrolyzates were quantified by HPLC as described below. All experiments were conducted in triplicate.

Saccharification percentages were calculated as:

(1) $$\%\;Saccharification=\frac{Sugar\;released*Cest}{Amount\;of\;sugar\;in\;substrate}*100$$

where *C_est_* is a stoichiometric correction factor to express the increase in molecular weight during hydrolysis (0.9 for glucose, 0.95 for cellobiose, and 0.88 for xylose and arabinose) [32,33].

### 2.7. Modelling of Enzymatic Hydrolysis

The kinetics of sugars released were modelized by Holtzapple empirical equation:

(2) $$\%\;CGC_{t}=CGC_{max}\cdot \frac{t}{t+t_{1/2}}$$

where *CGC_t_* is the cellulose to glucose conversion (%) at each time, *CGC_max_* is the maximum cellulose to glucose conversion predicted by model, *t* is the enzymatic hydrolysis time (h), and *t*_1/2_ is the time needed to reach the half of *CGC_max_*. The model of xylan and arabinan conversion were performed with the same equation.

### 2.8. Analytical Methods

#### 2.8.1. Determination of Enzymatic Activities

Xylanase activity was obtained by determining the released sugars from 1% (*w*/*v*) xylan from beechwood (Sigma Aldrich, Sant Louis, Mo, USA) prepared in 50 mM sodium citrate buffer pH 4.8, with incubation at 50 °C for 15 min [34].

CMCase (endo-β-1,4-glucanase) activity was obtained by determining the released sugars from 2% (*w*/*v*) carboxymethylcellulose (CMC) prepared in 50 mM sodium citrate buffer pH 4.8, with incubation at 50 °C for 30 min [35].

The two enzymatic reactions were stopped by the addition of dinitrosalicylic acid and boiling 5 min, and then the samples were cooled. The corresponding reducing sugars released in the reaction mixtures were quantified by absorbance measurements at 540 nm and further conversion to concentrations by using a standard calibration curve [36].

The β-glucosidase activity was measured using the method described by Leite et al. [19]. β-glucosidase activity was determined using p-nitrophenyl-β-D-glucopyranoside (PNG) as a substrate incubated at 50 °C for 15 min with a suitable dilution of enzyme in 50 mM sodium citrate buffer pH 4.8. The reaction was stopped by the addition of 1 M of Na_2_CO_3_. Finally, the absorbance was measured at 400 nm to determine the p-nitrophenol released.

One unit (U) of enzymatic activity corresponds to 1 µmol of xylose/glucose/p-nitrophenol released per minute, under the assay conditions.

The synergism factor (SF) of the two enzyme extracts used was calculated by the following equation:

(3) $$SF=\frac{m_{sj}}{m_{CLj}}$$

where *m_sj_* is the degree of glucan, xylan, or arabinan hydrolysis of mixture *j*, while *m_CLj_* is given by:

(4) $$m_{CLj}=\sum_{i=1,2}r_{ij}m_{i}$$

where *r_ij_* is the volume ratio of enzymes *i* in mixture *j* and m_i_ is the degree of glucan, xylan, or arabinan hydrolysis of cellulase *i*.

#### 2.8.2. Analysis of Sugars

Quantification of sugars (glucose, cellobiose, xylose, and arabinose) was done by HPLC (Agilent, model 1200, Palo Alto, CA, USA) equipped with a refractive index detector and an Aminex HPX-87H ion exclusion column (Bio Rad 300 mm × 7.8 mm, 9 m particles). The elution program was conducted during 23 min at 50 °C with a flow rate of 0.6 mL/min of 3 mM sulfuric acid [34].

### 2.9. Statistical Analysis

All data were compared by analysis of variance (ANOVA) and principal components analysis was performed with Statgraphics Centurion XVI.I software using Tukey’s test at a significance level of *p* < 0.05 to determine statistically significant differences.

## 3. Results

### 3.1. Enzymes Production by SSF

**Figure 1:**
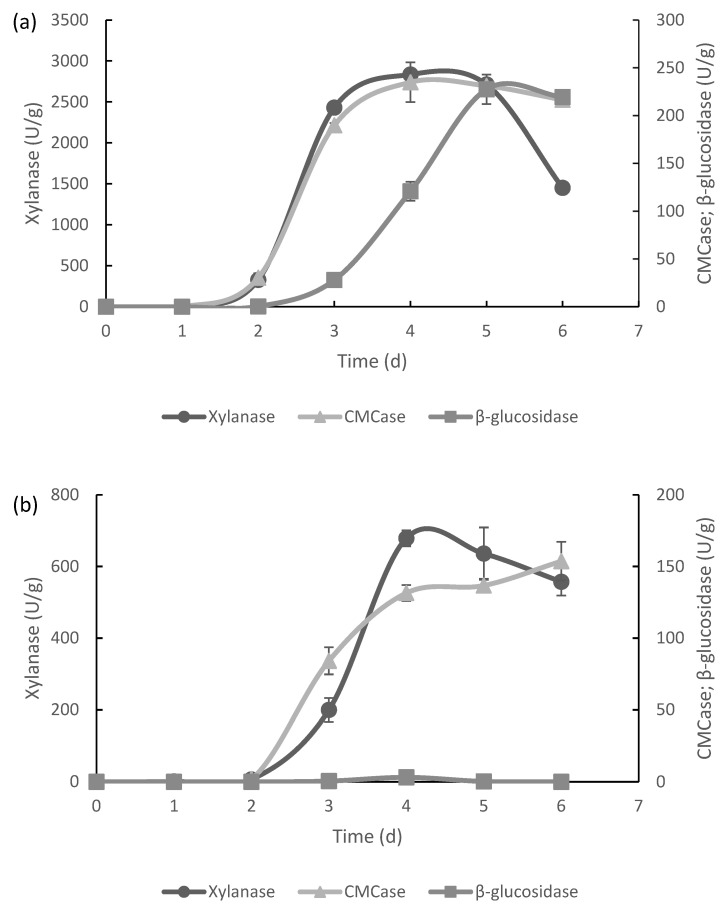
Time course of enzyme production by *A. brasiliensis* (**a**) and *T. reesei* (**b**) under solid-state fermentation.

The crude BSG was used as substrate to produce cellulolytic enzymes with *Aspergillus brasiliensis* CECT 2700 and *Trichoderma reesei* CECT 2414 by solid-state fermentation. Time course of enzymatic production using *A. brasiliensis* is shown in Figure 1a. The evolution of enzymatic activity during fermentation follows the trend of the typical profile described by [37]. The production of enzymes was detected after 2 days of SSF. The maximum xylanases production by *A. brasiliensis* was achieved between days 3 and 6 (2835.12 U/g). After 5 days, the production of enzymes decreased significantly (*p* < 0.05) to 1451.80 U/g. The maximum CMCase activity was 234.90 U/g on day 4, decreasing slightly in the following days. However, the CMCase concentration obtained on day 5 was not significantly different (*p* > 0.05) from that obtained on day 4. Regarding β-glucosidases, the maximum activity was obtained on day 5 (227.50 U/g) followed by a non-significant (*p* > 0.05) decrease at the end of the incubation.

These values were higher than those reported by Leite et al. [19] when performing SSF in BSG with related strains such as *Aspergillus niger* CECT 2088 with 246.41 U/g of xylanase, 51.35 U/g of CMCase, and 93.66 U/g of β-glucosidases or with *Aspergillus niger* CECT 2915 with 290.55 U/g of xylanase, 57.81 U/g of CMCase, and 3.98 U/g of β-glucosidases, which supports the suitability of this microorganism. De Souza Falcão et al. [38] concluded that *A. brasiliensis* is a good producer of cellulases, reporting a production of 39.85 U/g of CMCase using cupaçu residue as substrate. Moran-Aguilar et al. [13] reported a xylanase production of 2279.99 U/g of BSG after 7 days of fermentation using the same strain; this slightly lower value agrees with these data since we can observe the progressive decrease in activity of this enzyme from day 4. On the other hand, the xylanase activity was lower than that previously reported, 3152.39 U/g of BSG, performing the SSF in a 7 L horizontal drum bioreactor [32]. However, the β-glucosidases activity in this study was considerably higher than those reported by Moran-Aguilar et al. [13] (32.62 U/g) and Outeiriño et al. [32] (19.02 U/g).

Regarding the SSF of BSG with *T. reesei* (Figure 1b), it can be noted that the xylanase production peaked at 678.83 U/g obtained on day 4 (*p* < 0.05). The maximum CMCase activity was detected on day 6 with 153.75 U/g, and this enzyme concentration was not significantly different (*p* > 0.05) from the levels obtained on days 4 (131.50 U/g) and 5 (136.71 U/g). *T. reesei* was a poor β-glucosidases producer because the activity of this enzyme was only 3.15 U/g. 

It is known that *T. reesei* is a good producer of cellulases but also of xylanases. Kar et al. [39] found a xylanase production of 219 U/g in SSF of wheat bran with *T. reesei* SAF3. A three-fold increase in production was achieved in this study, supporting the importance of the substrate in SSF. The CMCase activity was much higher compared to that reported by Darabzadeh et al. [40] using the same strain grown on rice residues (0.553 U/g). On the other hand, Ben Taher et al. [41] reported activities of 41.8 U/g and 9.2 U/g for CMCase and β-glucosidases respectively, when performing SSF with *T. reesei* on potato peels residues, which shows the influence of the substrate in these processes.

In summary, *A. brasiliensis* showed the maximum xylanase, CMCase, and β-glucosidases after 5 days of SSF.

### 3.2. Composition of Raw and Pretreated Biomass

To facilitate the enzymatic hydrolysis of lignocellulosic materials, it is necessary to carry out a pretreatment to eliminate the causes of material recalcitrance such as a compact structure or a high lignin content [42]. For this purpose, in recent years, bioderived ionic liquids have stood out as a green and effective treatment [23,43].

In this case, BSG was treated with the IL [N_1112_OH][Gly] as described in a previous work. Briefly, BSG was treated with the IL at 90 °C for 16 h and mixed with a water/acetone solution, giving rise to a carbohydrate-rich material (CRM). CRM is formed by the combination of the materials obtained after 5 cycles of IL use, considering that IL can be recovered and efficiently reused for delignification of BSG during 5 cycles [31].

Table 1 shows the chemical characterization of crude BSG and CRM samples after pretreatment with IL [N_1112_OH][Gly]. In this case, the treatment with IL [N_1112_OH][Gly] achieved a drastic reduction in lignin (62.50%) in accordance with the data previously reported by [31] with delignification rates between 71–60%. These data agree with those published by other authors such as Hou et al., 2012 [44] with a delignification of 59.90% in rice straw, and Pakdeedachakiat et al. [45] with 60% of lignin reduction in mulberry stem. Because of this reduction in lignin, there is a considerable increase in carbohydrate fractions, being 1.6 times for glucan, 1.8 times for xylan, and 1.1 times for arabinan, with recoveries of these fractions of 52.4, 35.5, and 27.5% respectively.

### 3.3. Enzymatic Hydrolysis of CRM

One of the most ecological and profitable alternatives to obtain fermentable sugars from biomass is the enzyme-catalyzed hydrolysis of CRMs. The conditions for the saccharification of the material were selected according to previous works [32,46]. Additionally, Darabzadeh et al. [38] observed optimal temperatures for enzymatic hydrolysis with the enzymes of *T. reesei* CECT 2414 of 50 °C and a pH range of 4–5. In former works it was observed that enzymes produced by *A. brasiliensis* were suitable for saccharification of this type of material, despite not operating under optimal conditions [32].

#### 3.3.1. Effect of Solid and Enzyme Load

In a previous work, Paz et al. [10] determined the conditions for the enzymatic hydrolysis of BSG and pretreated BSG with NaOH using the enzymatic extract of *A. brasiliensis*. However, when the hydrolysis of BSG pretreated by IL [N_112_OH][Gly] was catalyzed by the enzymes produced by *A. brasiliensis* and conducted with a 1:10 solid–liquid ratio, the saccharification was low, with the formation of a mucilaginous suspension that made the access of enzymes to the substrate difficult.

Due to this, enzymatic hydrolysis tests were performed with higher solid–liquid ratios (1:30 and 1:60 *w*/*v*) using the previously obtained enzymatic extracts of *A. brasiliensis* and *T. reesei* diluted at different proportions with citrate buffer pH 5. Table 2 shows the results obtained from the saccharification of CRM with the *T. reesei* extract.

Generally, when the solid–liquid ratio 1:30 (*w*/*v*) was used, the hydrolysis with *T. reesei* extract provided poorer results in all fractions compared to the solid–liquid ratio 1:60 (*w*/*v*). This is due to the greater amount of free water improved the diffusion and action of enzymes in the enzymatic hydrolysis [9].

Regarding enzyme load, the best results were obtained using the higher enzyme load (60 mL/g BSG). However, it should be noted that glucan was released as cellobiose, as happens in all cases when using the *T. reesei* extract, meaning an incomplete saccharification of glucan into glucose. The remarkable cellulase and CMCase activities of the extract, which cause high cellulose hydrolysis, can explain this behavior, but lacking adequate levels of β-glucosidases, the cellobiose bonds are not broken and, therefore, the reaction does not end in obtaining a solution rich in glucose. Chen et al. [47] obtained a 65.9% hydrolysis yield of corn straw by applying enzymes from *T. reesei*, releasing glucose, xylose, and arabinose, detecting feedback inhibition by the accumulation of cellobiose due to the scarce β-glucosidases activity. Similarly, Chen et al. [48] obtained a 69.8% hydrolysis yield in corn cob. Other studies achieved a similar hydrolysis yield of glucose (78%) with lower solid load (15%, *w*/*s*) when BSG was pre-treated by chemical treatments [49]. However, two sequential chemical treatments with sulfuric acid were performed, which are more polluting than the treatment with ionic liquids [50]. Physical pretreatments with microwaves achieved a slightly lower yield (70%) of glucose with low solid load (5%, *w*/*v*) [51]. In addition, this thermal treatment may induce inhibitory products such as phenolic compounds and furfural [50].

When using the extract of *A. brasiliensis* (Table 3), as in the previous case, it is concluded that a higher solid–liquid ratio (1:60 *w*/*v*) led to a more efficient hydrolysis since the enzymes have greater mobility and easy access to the substrate. The best results were obtained when using 40 mL of the enzymatic extract. Contrary to the previous case, the hydrolysis catalyzed by the extract of *A. brasiliensis*, no cellobiose residues were obtained and only glucose was released into the medium caused by the hydrolysis of the glucan fraction. The better hydrolysis yield obtained when using 40 mL of enzyme extract instead of 60 mL may be due to the fact that the CMCase–β-glucosidase ratio produces a better synergy.

Several works have studied the synergy with different proportions of cellobiohydrolase and endoglucanase, concluding that the characteristics of the enzyme, the assay conditions, and the characteristics of the substrate influence synergistic actions [52].

Previously, the feasibility of using the enzymatic extract obtained by SSF from *A. brasiliensis* on BSG residues after SSF was already demonstrated [32]. It was possible to hydrolyze 24.12% of glucan, 54.37% of xylan, and 57.04% of arabinan under suboptimal conditions. Paz et al., 2019 [10] also hydrolyzed BSG treated with [N_1112_OH][Gly] using the enzymatic extract of *A. brasiliensis,* reporting conversions of 29.62% of xylan and 30.83% of arabinan showing evident signs of an inefficient hydrolysis when it was carried out with a high-solid load.

Comparing the two best conditions for both extracts, the *A. brasiliensis* extract led to better results of sugars released.

#### 3.3.2. Effect of Enzymatic Extracts Mixtures

Although the glucose released was higher with the *A. brasiliensis* extract than with *T. reesei* extract, this increase does not represent a significant improvement (*p* > 0.05). This leads to the combined use of the two extracts to solve the deficiencies that they may have and favor synergistic actions. In this sense, Chen et al. [47,48] obtained better results when hydrolyzing corn cob by combining enzymes from *T. reesei* with enzymes from *A. niger*, since the latter contains greater amounts of β-glucosidases.

Therefore, the extracts were combined in different *Aspergillus*/*Trichoderma* ratios (*v*/*v*): 1:1 (denoted as MA), 1:2 (MB), 2:1 (MC), and 2.5:0.5 (MD). The hydrolysis of CRMs was carried out keeping fixed the solid–liquid ratio of the reaction at 1:60 (*w*/*v*). The results of the hydrolysis of CRMs using these enzymatic cocktails are shown in Table 4.

Similar results were obtained with enzymatic cocktails MA, MB, and MC, hydrolyzing in all cases around 60% and 50% of glucan and xylan, respectively, not showing significant differences between them (*p* > 0.05). Only in the fraction arabinan were significant differences found, showing that MB produced the worse results, and that MA and MC were significantly the same in this regard. The results obtained with the MD extract were significantly better (*p* < 0.05) for the saccharification of glucan and xylan with values of 84.67% and 71.21% respectively, while the hydrolysis of arabinan with MA and MC provided similar results.

The synergism between two enzymatic extracts was calculated (Table 4). A synergism effect was observed for all mixtures to hydrolyze glucan, xylan, or arabinan except for mixtures MA and MB for glucan and mixture MB for arabinan. The highest value of synergism was achieved with mixture MD for all polysaccharides.

Although the cellulase activity of the MD extract is significantly lower than that of the MC extract, the improvement in glucan saccharification can be attributed to a higher β-glucosidase activity in MD. MD also has a slightly higher xylanase activity, which can cause a synergistic effect, by hydrolyzing the xylan bound to cellulose, thus allowing its hydrolysis by cellulases [53].

**Figure 2:**
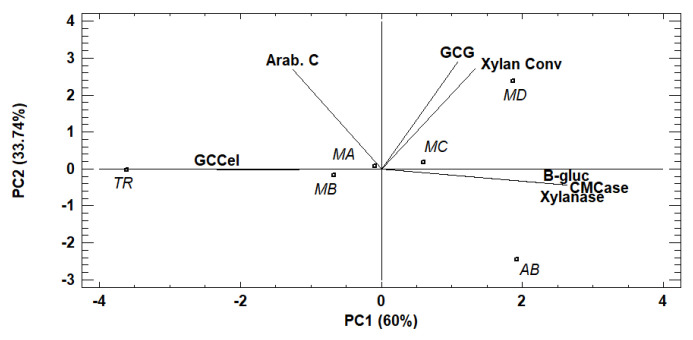
Principal components analysis biplot of effect of enzymatic mixtures on saccharification. TR: *Trichoderma reesei*, AB: *Aspergillus brasiliensis*; MA: AB/TR (1:1); MB: AB/TR (1:2); MC: AB/TR (2:1); MD: AB/TR (2.5:0.5); Arab. C: arabinan conversion; GCG: glucan conversion to glucose; GCCel: glucan conversion to cellobiose; Xylan conv.: xylan conversion.

A principal components analysis (PCA) was applied to evaluate the effect of enzymatic extracts and their mixtures on the saccharification of CRM (Figure 2). The analysis explained 93.7% of the variance of results. As it can be observed, the first component (PC1) positively characterized the enzyme activities, the GCG (glucan conversion to glucose), and the xylan conversion, and negatively characterized the arabinan conversion and glucan to cellobiose conversion. According to the plot, the MD and AB enzymatic extracts were positively correlated with GCG and xylan conversion, however the other mixtures and TR extract were not correlated with high GCG and xylan conversion.

In conclusion, the use of the *A. brasiliensis* extract with smaller amounts of the *T. reesei* extract has a positive effect that leads to more efficient hydrolysis, for which the condition selected to continue the study was a solid–liquid ratio 1:60 (*w*/*v*) using the enzymatic cocktail MD.

**Figure 3:**
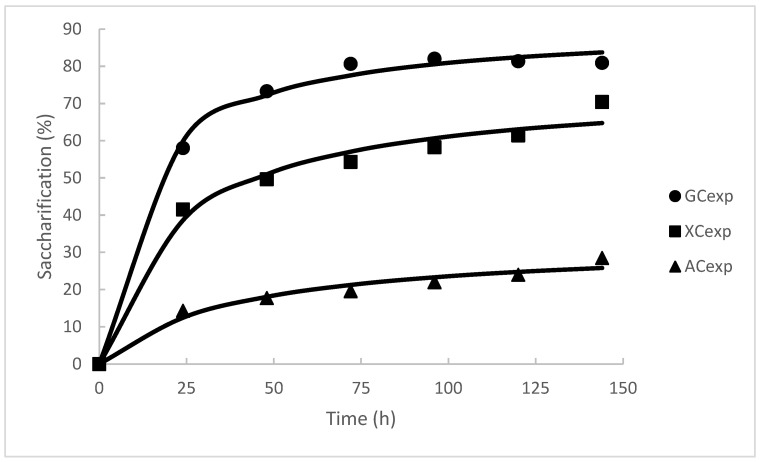
Modelling of enzymatic hydrolysis by extract MD. GCexp: glucan experimental conversion; XCexp: xylan experimental conversion; ACexp: Arabinose experimental conversion.

Next, the kinetics of the reaction were studied under the selected conditions to evaluate the evolution of saccharification. As observed in Figure 3, the saccharification of glucan increased rapidly to 58.04% during the first 24 h of incubation, but after this time, the saccharification rate began to decrease until reaching a value of 80.67% at 72 h. In the following days, the saccharification of the polysaccharide did not show significant changes (*p* > 0.05). The xylan was hydrolyzed at a higher rate in the first 24 h, then its hydrolysis increased almost linearly until reaching a final value of 70.40% at the end of the incubation (144 h). The hydrolysis of arabinan showed a profile like that observed for xylan, but with a more moderate increase, reaching a maximum of 28.49% at 144 h.

The release of glucose (R^2^: 0.9993), xylose (R^2^: 0.9969), and arabinose (R^2^: 0.9943) showed a good fit to the model (Equation (2)). Table 5 summarizes the values of *t*_1/2_ and *SC_max_*. The maximum glucose released described by the model was 91.02% (see Table 5). The *t*_1/2_ to release glucose was lower than the *t*_1/2_ to hydrolyze xylan and arabinan. Although the amounts of xylose and arabinose released were greater as time increased, the release of glucose was considered the most relevant factor to select the most appropriate hydrolysis time since this sugar is preferentially consumed by the lactic acid bacteria that will be used to produce bacteriocins and biosurfactants [54,55]. For this reason, a time of 72 h was selected to perform the hydrolysis of CRM, because at this time the saccharification percentages of glucan, xylan, and arabinan obtained were 80.68%, 54.29%, and 19.58%, respectively.

To corroborate this study, a hydrolysis of raw BSG under the same conditions was conducted. After stopping the reaction at 72 h, BSG hydrolysis percentages of 41.63 ± 0.36%, 36.18 ± 1.06%, and 52.11 ± 0.48% were obtained for glucan, xylan, and arabinan, respectively. This means that pretreatment with IL improves the hydrolysis of glucans by 51.59% and xylan by 66.62%, while the fraction of arabinan released 37.56% more when using raw BSG.

## 4. Conclusions

The results obtained in this work indicate that BSG is a suitable material for a biorefinery process. BSG was an appropriate substrate to obtain enzymatic cocktails by *Aspergillus brasiliensis* CECT 2700 and *Trichoderma reesei* CECT 2414 under SSF. A material with a high carbohydrate content and more easily hydrolyzable was achieved by pretreating the BSG with the IL [N_112_OH][Gly]. A mixture of the enzymatic extracts in the ratio 2.5:0.5 *Aspergillus*/*Trichoderma* (*v*/*v*) achieved an efficient hydrolysis of CRM.

## Figures and Tables

**Table 1:** Composition of BSG and CRM expressed as g per 100 g of dry solid.

Component	BSG	CRM
Moisture	6.82 ± 0.04	6.84 ± 0.35
Ashes	2.87 ± 0.04	3.45 ± 0.02
Klason lignin	11.98 ± 0.02	7.59 ± 0.41
Soluble lignin	7.32 ± 0.03	1.88 ± 0.03
Extracts	14.00 ± 0.33	n.d.
Glucan	27.77 ± 0.35	45.73 ± 0.90
Xylan	16.46 ± 0.51	30.72 ± 0.34
Arabinan	8.44 ± 0.13	9.70 ± 0.11
Acetyl group	3.02 ± 0.04	n.d.

n.d.: not detected; BSG: brewery spent grain; CRM: carbohydrate rich material.

**Table 2:** Hydrolysis of CRM catalyzed by the enzymatic extract of *T. reesei*.

LSR	1:30 (*w*/*v*)			1:60 (*w*/*v*)		
EE (mL/g BSG)	10	20	30	20	40	60
Glucose (%)	12.75 ± 1.34	23.14 ± 0.19	23.51 ± 0.54	22.09 ± 0.49	42.76 ± 2.30	48.03 ± 2.34
Cellobiose (%)	34.19 ± 2.64	23.62 ± 1.30	37.64 ± 0.44	24.30 ± 0.66	30.09 ± 0.06	30.09 ± 0.06
Glucan (%) *	48.20 ± 3.40	46.76 ± 1.50	61.58 ± 0.12	46.38 ± 0.16	71.47 ± 3.11	78.11 ± 2.28
Xylan (%)	12.51 ± 0.93	23.52 ± 1.28	30.15 ± 0.58	17.94 ± 1.95	34.24 ± 1.43	37.92 ± 2.96
Arabinan (%)	14.49 ± 1.19	18.75 ± 0.65	19.80 ± 0.40	17.90 ± 1.06	25.38 ± 2.19	31.64 ± 1.27

* Glucan (%) corresponds to the sum of Glucose (%) and Cellobiose (%); EE: enzymatic extract; LSR: liquid-solid ratio.

**Table 3:** Hydrolysis of CRM catalyzed by the enzymatic extract of *A. brasiliensis*.

LSR	1:30 (*w*/*v*)				1:60 (*w*/*v*)	
EE (mL/g BSG)	10	20	30	20	40	60
Glucose (%)	29.14 ± 1.35	39.74 ± 0.67	33.86 ± 0.97	39.82 ± 0.31	52.73 ± 0.47	42.81 ± 3.28
Cellobiose (%)	n.d.	n.d.	n.d.	n.d.	n.d.	n.d.
Glucan (%) *	29.14 ± 1.35	39.74 ± 0.67	33.86 ± 0.97	39.82 ± 0.31	52.73 ± 0.47	42.81 ± 3.28
Xylan (%)	33.39 ± 0.98	35.53 ± 0.75	30.25 ± 0.85	41.47 ± 0.62	46.23 ± 1.13	37.88 ± 2.42
Arabinan (%)	18.87 ± 0.00	19.42 ± 0.11	14.45 ± 1.04	23.12 ± 0.94	25.61 ± 0.67	20.16 ± 1.15

* Glucan (%) corresponds to the sum of Glucose (%) and Cellobiose (%). n.d.: not detected; EE: enzymatic extract; LSR: liquid-solid ratio.

**Table 4:** Saccharification of the CRM fractions using the enzyme extract mixtures and a solid–liquid ratio of 1:60 (*w*/*v*).

	MA	MB	MC	MD
Glucan (%)	58.89 ± 2.37 a	56.17 ± 4.33 a	60.65 ± 3.16 a	84.67 ± 0.77 b
Xylan (%)	47.40 ± 1.90 a	48.63 ± 0.00 a	50.45 ± 0.05 a	71.21 ± 3.13 b
Arabinan (%)	28.24 ± 0.68 ab	25.87 ± 0.25 a	28.36 ± 1.18 ab	31.39 ± 1.26 b
SF (Glucan)	0.97	0.85	1.11	1.74
SF (Xylan)	1.25	1.28	1.33	1.88
SF (Arabinan)	1.09	0.93	1.19	1.42

*Aspergillus*/*Trichoderma* ratios (*v*/*v*): 1:1 (denoted as MA), 1:2 (MB), 2:1 (MC), and 2.5:0.5 (MD). SF (synergism factor). Means within rows followed by the same letter are not significantly different at *p* > 0.05.

**Table 5:** Parameters of kinetic enzymatic model using the extract MD.

	*t*_1/2_ (h)	*SC_max_* (%)
Glucan conversion	12.48	91.02
Xylan conversion	22.56	74.92
Arabinan conversion	39.44	32.89

*t*_1/2_: time needed to reach the half of *CGC_max_*; *SC_max_*: maximum sugar conversion.

## Data Availability

The datasets generated for this study are available on request to the corresponding author.
